# Contextualising, Conceptualising and Constructing At‐Homeness: Towards an Integrative Framework for Residential Care

**DOI:** 10.1111/1467-9566.70230

**Published:** 2026-07-05

**Authors:** Jialiang Cui, Jialing Wu

**Affiliations:** ^1^ Department of Social Work The Chinese University of Hong Kong Shatin New Territories Hong Kong

## Abstract

The concept of at‐homeness has been increasingly adopted in residential care to describe a metaphorical sense of being “at home”. Within a sector often criticised for displacing residents from familiar environments, at‐homeness has come to symbolise the ideal experiential outcome of care. Yet, despite a growing body of work enriching our understanding of what at‐homeness entails, existing conceptualisations tend to overlook the processes through which at‐homeness is personally and multiply constructed and are shaped predominantly by studies of older adults in long‐term care contexts. To address these limitations, a qualitative study, employing a user‐involved approach, was conducted in Hong Kong, incorporating in‐depth interviews with 60 residents of mental health residential services and ethnographic observations across three hostels. Analysis shows that at‐homeness is shaped through boundary‐setting and bonding between the self and three arenas—social relations (“people”), material environments and temporal orientations. Residents interpreted core qualities of at‐homeness across personal, relational, material and temporal dimensions and their constructions were guided by distinct logics involving self‐appraisal, temporal integration, relational attunement and socio‐cultural grounding. The study proposes an integrative framework that reframes at‐homeness as a dynamic, negotiated process, offering new insights for advancing theory and strengthening methodological and practice approaches in residential care.

## 1 Introduction

The field of residential care is rife with contestations, reflecting deep tensions between care and autonomy in contemporary welfare systems. On the one hand, residential care offers indispensable accommodation and support for some children, older people and individuals with specific conditions (e.g., homelessness, disabilities or terminal illnesses), particularly when they lack the capacity or resources to live independently. In many urban regions worldwide, the demand for publicly funded residential care significantly exceeds available supply (Rahman et al. 2022). Yet, paradoxically, residential care continues to be framed as a suboptimal or even undesirable option. It has long been criticised for undermining autonomy, eroding self‐esteem and fostering loneliness (Cooney et al. 2009; Krotofil et al. 2018). Some policy discourses also portrayed it as inconsistent with the ideal of ageing in place (El‐Bialy et al. 2022) or as a system that deprives children and people with disabilities of opportunities to live in the community for social inclusion (Rosenthal 2021).

In response to these tensions, a strong and persistent trend has emerged over recent decades to infuse the idea of home into the delivery of residential care. Across diverse contexts, such as palliative and hospice care (Saarnio et al. 2016), assisted living and nursing homes (Cater et al. 2022; Lewinson et al. 2012) and homelessness shelters (Hoolachan 2020; Söderqvist et al. 2016), the creation of home has been framed as a shared aspiration among residents. The experience of home has increasingly been conceptualised as “at‐homeness” (sometimes used interchangeably with “homeliness” or “homelike”), a term introduced by Seamon (1979, 70) to capture the subtle, often unarticulated experience of being at ease and familiar within one's everyday world—contrasted with the feelings of being “in transit”, “not at home” or “out of place”. This conceptual shift has generated a substantial body of research examining how care environments and practices seek to approximate, assess and operationalise experiences of home in residential care (Molony et al. 2007; Öhlén et al. 2014; Saarnio et al. 2016; Ausserhofer et al. 2016; Fleming et al. 2017).

Despite the burgeoning interest in at‐homeness, critical questions have come to the fore. What does this experience of home mean for people living in unconventional residential circumstances? How is it constructed, traversed and co‐constituted across changing times, places, relationships and social contexts? These questions are key to bridging the idea of at‐homeness with its lived expression in care settings. However, the conceptual sophistication developed around the notion of home has not been adequately integrated into research on residential care (Fleming et al. 2017; Lovatt 2018), potentially leading to fragmented or ineffective attempts to translate the concept into interventions. This paper examines the construction of at‐homeness in the context of mental health transitional accommodation—a continuum of short‐ and medium‐term residential services supporting the housing and recovery of people with mental health issues in Hong Kong—by focussing on how residents construct and negotiate a sense of home within such settings.

### 1.1 Home and At‐Homeness in Residential Care

The breadth and depth of scholarship on home is remarkable, conceptualising home in myriad ways as place, feeling, memory, experience, identity and imagination (Somerville 1992; Blunt and Dowling 2022; Chambers 2020; Mallett 2004). Conventionally, understandings of home have been closely tied to the materiality of dwelling, particularly the private domestic sphere distinguished from public space and associated with stability, security and cherished experience. This orientation is reflected in the prominence of housing studies within home scholarship, which examine architectural design and domestic arrangements alongside the sociological, economic and political dimensions of housing provision (Blunt and Dowling 2022). With the rise of social constructionist paradigms in the late twentieth century, however, this framing was unsettled. Greater emphasis was placed on the symbolic, relational and affective dimensions of home that speak to one's fundamental ontological needs (Boccagni 2022; Molony 2010; Roster et al. 2016). As Easthope (2004, 36) notes, “While home may be located, it is not the location that is home”. Duyvendak (2011) further systematised the concept by classifying core elements of home into familiarity, haven (e.g., the experience of safety, comfort and privacy) and heaven (e.g., the formation of public identity through attachment to place).

Yet both material and existential conceptualisations have been critically interrogated by feminist scholars for their tendency to romanticise home, thereby obscuring the harm, constraint and exclusion enacted within domestic spaces (Brickell 2012). The critical perspective on home destabilises the taken‐for‐granted association between home, intimacy and belonging, instead foregrounding the contested character of dwelling. In sociology, home is increasingly understood as simultaneously spatial and emotional (Boccagni and Kusenbach 2020), highlighting the everyday practices through which people negotiate belonging, construct identity and reconfigure what it means to dwell. Much recent research underscores this multiplicity of lived experience of home, demonstrating that even transient, commercial and public environments (e.g., student dormitory, Airbnb and restaurants) may become incorporated into individuals' meaningful constructions of home (Blunt and Dowling 2022; Miranda‐Nieto and Boccagni 2020; Pechurina 2025).

Extending these insights to residential care, delinking home from static materiality and idealised domesticity foregrounds at‐homeness as a pivotal orientation for legitimising and improving residential care, a sector long subject to critique for displacing service users from familiar environments and disrupting their sense of home. Building on Seamon's (1979) original formulation, at‐homeness has been adapted in health and social care as “a feeling of being metaphorically at home” (Öhlén et al. 2014, 1) and has come to denote the optimal experiential outcomes of residential care (Hilli and Eriksson 2019). Zingmark et al. ’s (1993) early study of people with dementia in group dwellings emphasised that at‐homeness must be integrated into care philosophy, even where physical environments are already designed to appear homelike. Since then, scholarly and practice interest, particularly in nursing and Scandinavian research, has expanded to examine both the meanings of at‐homeness and the conditions under which it could be fostered in residential care and during transitions into such settings (Saarnio et al. 2019; Søvde et al. 2022).

The complexity of home scholarship, together with burgeoning interest in at‐homeness, has prompted a range of attempts to essentialise its meanings, which we encapsulate into two broad strands. The first seeks to articulate the contextual dimensions that shape the making and unmaking of at‐homeness in residential care, extending the conventional home scholarship that understands home as a physical place. For instance, Zingmark et al. (1993) identified six interdependent and interrelated dimensions of at‐homeness, relating to self, others, time, objects, events and place. Falk et al. (2013), drawing on the experiences of older adults undergoing inter‐institutional relocation, proposed a simplified framework emphasising three dimensions of environments in which at‐homeness is constituted: attachment to place, spaces (social relations) and contexts extending beyond the institution.

The second strand focuses on the existential dimensions of at‐homeness rather than the specific contexts where these experiences unfold. For example, Molony et al. (2007) advanced a four‐component framework encompassing: (1) separation, denoting personal space and control; (2) connection, referring to relationships and continuity; (3) dynamic transitions, emphasising care and the exchange of energy; and (4) atmosphere, described as warmth and belonging. Similarly, Öhlén et al. (2014), in their review of the conceptual development of at‐homeness, condensed the notion into three aspects: safety, connectedness and centredness. Undeniably, these conceptual efforts have contributed to embedding at‐homeness into residential care practices, exemplified by the development of the *Experience of Home Scale* (Molony et al. 2007), which operationalises these qualities to support both implementation and evaluation.

What has largely escaped attention in this conceptual development is the integration of the critical perspective on home, as evidenced by the lack of investigation of the very process through which the notion of at‐homeness is individually and multiply constructed. Although many scholars have sought to clarify the essential components that an ideal level of at‐homeness might encompass, critical questions remain unanswered. Why are these particular components invoked in one's imagination of experiencing home? How are they positioned in relation to one another and broader social realities? And must all of them be present for the experience of home to be fully realised? This line of inquiry is important, given a trend to associate the concept with a sense of wholeness and holism (e.g., Molony 2010; Saarnio et al. 2016). Although this trend may contribute to translating the notion into residential care, for example, through developing standardised instruments to measure at‐homeness (Molony et al. 2007), it may also inadvertently perpetuate a normative ideal that obscures residents' agentic subjectivities and the authentic needs underpinning their personal constructions.

### 1.2 At‐Homeness Beyond Elderly Residential Care

Research on at‐homeness in residential care has developed most extensively within studies of older people living in long‐term care settings. Although this body of work has generated valuable insights into the conceptual development of at‐homeness, its strong concentration on later‐life residential care has meant that at‐homeness is often examined within relatively stable institutional contexts and through age‐specific subjectivities. Yet core human needs are socially negotiated and contextualised across age, life stage and social roles (Buijs et al. 2021). Experiences and meanings of home, therefore, may be constructed in variable ways across the life course and residential contexts (Klaassens and Meijering 2015).

Beyond elderly long‐term care, a growing body of literature has examined experiences related to home and homemaking in other accommodation contexts, such as youth hostels, homeless shelters and residential settings for people with special needs. Many mental health studies explored residents' lived experiences in residential services, with aspects of these experiences subsequently interpreted through the lens of home. For example, O'Donovan et al. (2019), in their study of people experiencing homelessness and mental illness, linked participants' feeling of “my place” at a hostel to a sense of home, whereas Krotofil et al. ’s (2018) systematic review of mental health accommodation research subsumed a range of positive experiential dimensions under the category of “home”. In research on temporary accommodation, scholars have often focused more directly on how a sense of home is produced in unconventional living environments. Primary attention has been given to material and organisational dimensions—such as fittings, fixtures, spatial layouts and institutional rules governing usage and access—and how these are designed and negotiated, shaping residents' identity and comfort whilst mediating everyday experiences through organisational authority (Harris et al. 2020; Hoolachan 2020; Parrott 2005; Hao et al. 2022). In addition, a recent study by Chinn et al. (2024) introduced the concept of “feeling at home” to people with intellectual disabilities living in group homes. Through photovoice, their experiences of at‐homeness were understood as multidimensional, encompassing personal, physical and social dimensions of home. Although these studies offer valuable insights into meanings and factors associated with feeling at home, they often sidestep a systematic examination of residents' own constructions of at‐homeness, thereby limiting understanding of how diverse imaginaries of home are negotiated and translated into everyday practices in these settings.

This paper addresses these gaps by directly examining constructions of at‐homeness in the context of mental health transitional accommodation in Hong Kong, where people with mental health issues constitute the largest disability group (Census and Statistics Department 2021). Transitional accommodation is the predominant form of residential care for this clientele in Hong Kong, comprising over 40 publicly funded hostels that accommodate approximately 1700 residents, with persistent calls for further expansion due to long waiting lists (Labor and Welfare Bureau 2018). These settings serve a relatively wide age range, beginning at age 15 with no upper limit. Although framed as transitional, the length of stay is not strictly regulated and some residents remain for extended periods, sometimes exceeding a decade. Most facilities operate as communal living environments housing between 20 and 80 residents, with 24‐h on‐site staffing, structured routines and institutional rules governing daily life, access and prohibited behaviours. Although residents are generally encouraged to pursue employment and community integration, physical and environmental restrictions may still be enforced, particularly when residents are physically unwell or experiencing mental instability. This combination of temporality, regulation and aspirational independence renders these settings a particularly complex and revealing context for examining at‐homeness.

This paper examines at‐homeness through three interrelated lenses: contextualising where and how it arises, conceptualising what it entails, and, more importantly, analysing how it is dynamically constructed from residents' own perspectives. By investigating not only what it means to feel at home but also how residents actively construct and negotiate this sense of at‐homeness within dynamic material, relational, temporal and socio‐cultural contexts, the paper aims to contribute to the development of a more fine‐grained, constructivist and integrative framework for understanding narratives of at‐homeness in residential care. Such a framework has the potential to enhance the practical relevance of this line of inquiry in health and social care, aligning it more closely with the ethos of person‐centred care.

## 2 Methods

To address the research aim, an in‐depth qualitative study was conducted between 2024 and 2025. Aligning with the principle of “nothing about us without us” in disability research (Charlton 1998), we adopted a user‐involved research approach in collaboration with the*Alliance of Persons in Mental Recovery of Hong Kong*. Seven members with lived experience of mental health residential care were invited to join the research team as an expert panel to advise on the study design, data collection and analysis. Drawing on their input, two qualitative methods were adopted: semi‐structured interviews and focused ethnography (Knoblauch 2005). Ethical approval for this study was granted by the research ethics committee of the Chinese University of Hong Kong (SBRE‐22–0195).

The semi‐structured interviews were conducted with 60 residents (aged 23–63) recruited from 17 hostels providing accommodation and support for people with mental illness. Each interview lasted around 60–90 min and incorporated two interactive exercises (i.e., drawing and card‐sorting) to facilitate participants' sharing. First, participants were invited to create a “rich picture” (Matthews 2014) of their ideal home. This exercise served as both an icebreaker and a reflective tool, allowing participants to express abstract ideas visually and to articulate meanings that might be difficult to convey verbally (Clark and Morriss 2017). Participants were then encouraged to interpret their drawings, explaining the content, use of colour, lines, forms and the origins of their imagined home.

The second activity, a card‐sorting exercise, explored personal meanings of at‐homeness. An initial set of seventeen cards representing the essences of at‐homeness was developed through a literature review and consultation with our expert panel. The cards were organised into three thematic dimensions: (1) personal features, denoting internal and self‐directed aspects (e.g., safety, freedom, identity); (2) relational attributes, referring to socially connected qualities (e.g., connectedness, care, respect); and (3) physical qualities, capturing material aspects (e.g., food, tidiness). Participants were invited to select or create cards that resonated with their drawing of home, interpret their choices in light of their lived experience and rank the cards by importance, explaining the reasoning behind the sequence. The interview guide was piloted with the panel members to ensure the process was intuitive and effective in eliciting participants' lived experiences. During each interview, interviewers took field notes to record non‐verbal cues and contextual observations.

Following the preliminary analysis of the interview data, two researchers conducted focused ethnography involving 2 weeks of observation at each of three distinct residential settings: a female‐only hostel located within a multi‐storey service complex, a male‐only hostel situated in a public housing estate and a stand‐alone mixed‐gender hostel. Data collection methods included participant observation, which involved engaging in daily training and outdoor activities, as well as conversations with residents, staff, family members and visitors. Through these ethnographic observations, the constructions of at‐homeness were brought to life and imbued with more vivid, nuanced meanings as they unfolded in everyday actions and exchanges.

For data analysis, all interview recordings were transcribed verbatim and the ethnographic data were recorded as detailed field notes; all personally identifiable names were replaced with common English pseudonyms to ensure anonymity. Data from these sources were systematically organised and analysed using thematic analysis (Braun and Clarke 2013). All transcripts and field notes were managed and coded using Delve software. The coding process began with a close reading of five transcripts to generate an initial set of inductive codes. From this, a preliminary coding protocol was developed through line‐by‐line analysis. After discussion and agreement among the research team, this coding framework was iteratively applied to the remaining dataset. The ethnographic data were analysed thematically and served both to enrich and to triangulate the interview findings, offering contextual insights into how at‐homeness was enacted and negotiated in daily life.

## 3 Findings

Analyses of the interview and ethnographic data reveal that at‐homeness in residential care is a dynamic, multi‐layered experience. The findings are presented in three parts. The first, *contextualising at‐homeness*, examines the arenas in which the sense of at‐homeness emerges. The second, *conceptualising at‐homeness*, identifies its core experiential meanings as they correspond to diverse contexts. The third, *constructing at‐homeness,* explores how residents create their own idea of at‐homeness by integrating needs, experiences and aspirations.

### 3.1 Contextualising At‐Homeness

The feeling of home does not exist in a void. Contextualising at‐homeness is pivotal to understanding how it is constructed and continually negotiated within residential care. Drawings, participant accounts and researcher observations revealed that the experience of home was imagined through boundary‐setting and bonding between the self and three interrelated arenas: “people”, material environments and time.

**FIGURE 1:**
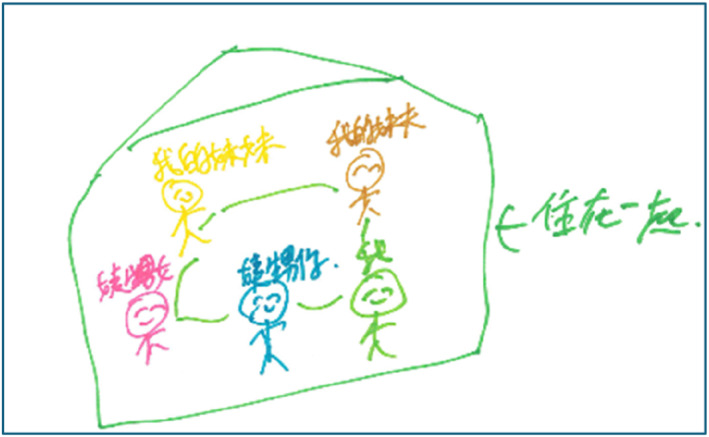
*Linda's* emphasis on family.

**FIGURE 2:**
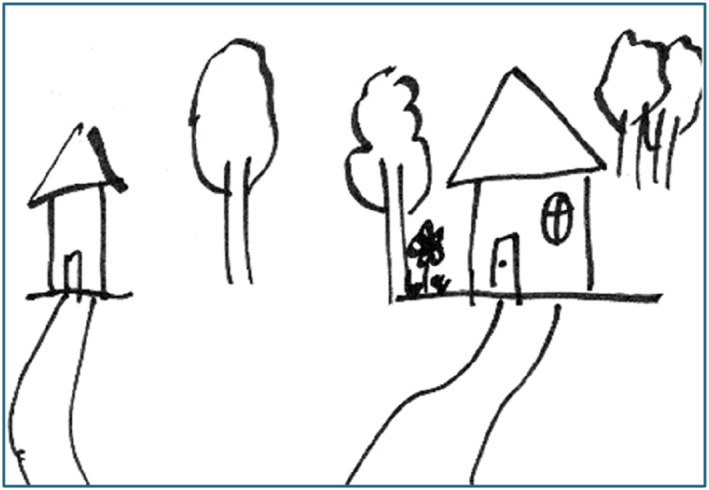
*Jeffrey's* emphasis on neighbours.

**FIGURE 3:**
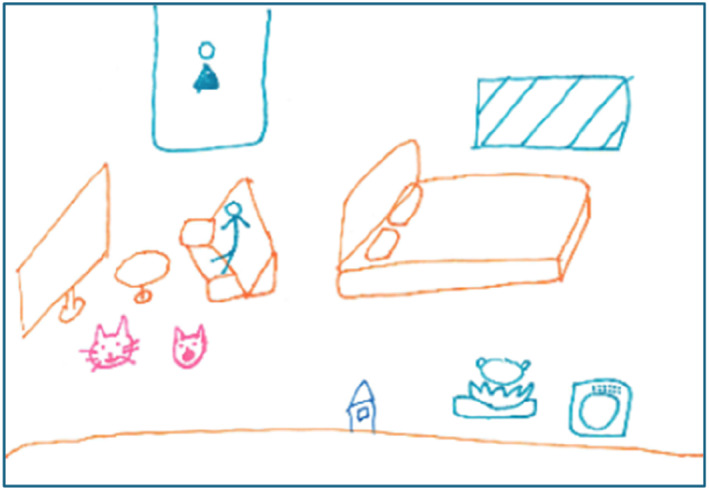
*William’s* emphasis on pets.

The relational arena of at‐homeness concerns the inclusion/exclusion of “people” in shaping the existential insideness of home. For some participants, home was defined by boundaries that protected personal autonomy. For example, *James,* a man in his late 40s who denied having any mental illness, described himself as being “forced” to live in a hostel. As he explained, “*home is for myself only—if a friend asked to stay, I'd rather lend him money to stay somewhere else.*” In contrast, others included selected individuals within their scope of home and some even equated home with this relational closeness. As *John,* a 28‐year‐old recent resident who lived by himself before admission, put it, “*living by myself doesn't make a home; family does.*” Furthermore, the category of “people” in some participant accounts extended beyond kinship to broader relations (e.g., neighbours, co‐residents and staff) and even pets, which several described as vital companions and sources of emotional bonding (Figures 1, 2, 3).

**FIGURE 4:**
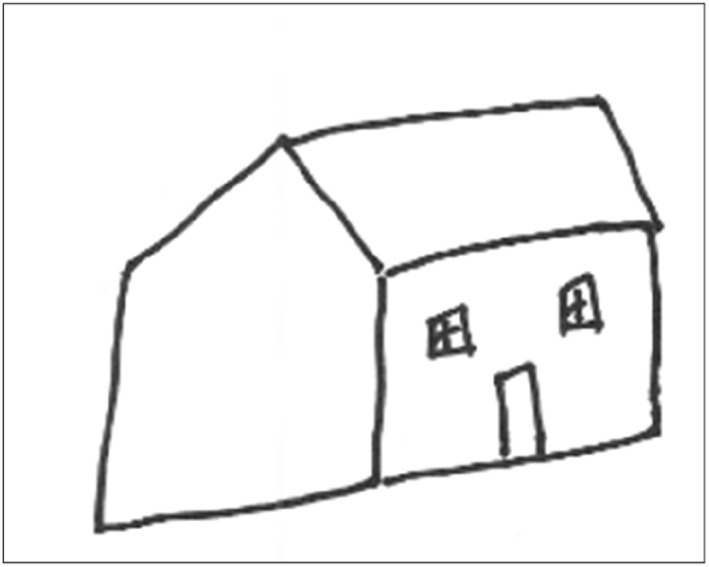
*Elizabeth's* drawing of a house.

**FIGURE 5:**
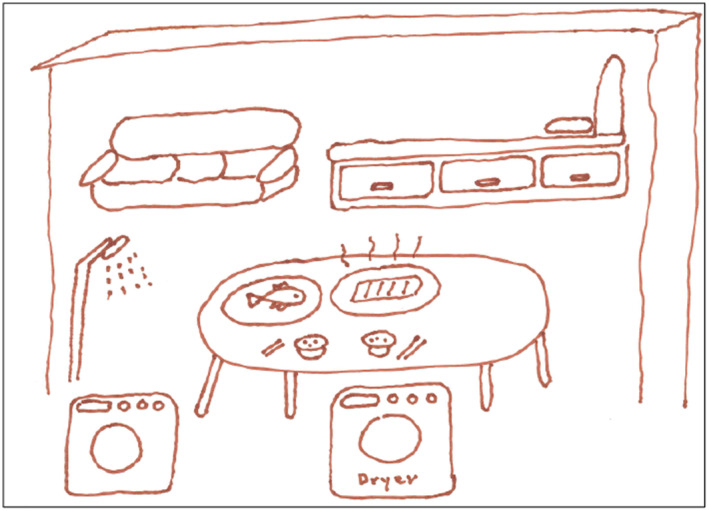
*Philip's* drawing of furniture.

**FIGURE 6:**
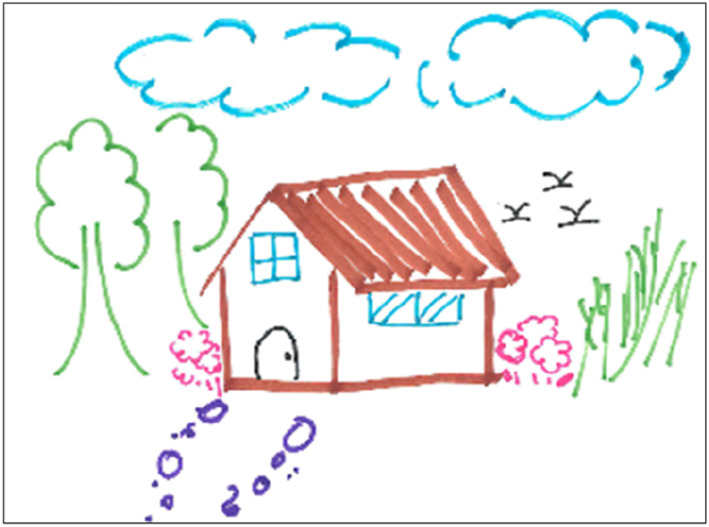
*Donna's* drawing of the surrounding environment.

**FIGURE 7:**
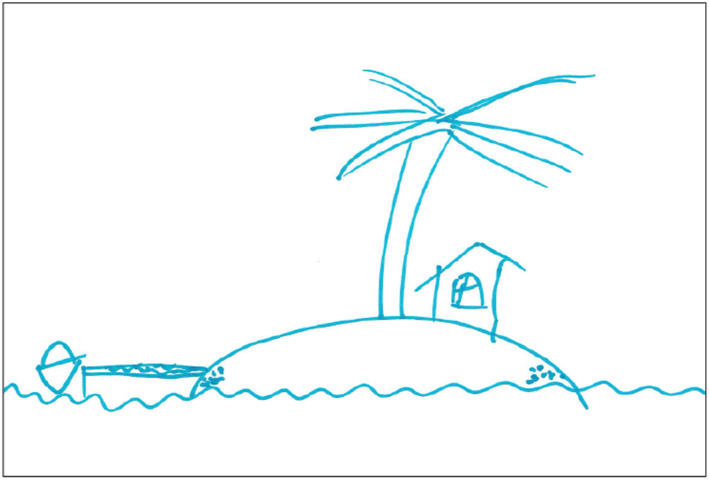
*Dennis's* drawing of imagined space.

In the material arena of at‐homeness, participants selected—and at times deliberately excluded—objects and places that either safeguarded the “people” within from perceived danger or fulfilled their everyday and existential needs. As illustrated in Figures 4, 5, 6, the material world of home encompasses physical dwellings (e.g., houses or apartments), the objects and furniture within them and the surrounding environment (e.g., trees, flowers). For some, it also extended to imagined spaces—a symbolic place where they felt most “at home” (e.g., a quiet island in Figure 7). This process of selection was not a simple juxtaposition of material elements within one's life world but an interactive bonding process through which materiality acquired personal and emotional significance. For example, as shown in Figure 5, the presence of steaming food or the choice of clothes dryer to adapt to Hong Kong's humid weather embodied residents' lived needs, past experiences and situated understandings of their environments.

Furthermore, the idea of home did not necessarily refer to a single or fixed location/object, particularly within residential settings where residents' movements are not confined to one site. Instead, places and spaces (e.g., hostels, parents' homes or community venues) could be intertwined in residents' constructions of home. For instance, at the first ethnographic site, we met *Bo*, a man in his 50s who expressed high satisfaction with the hostel and had chosen to return to live there for a second time. Yet his sense of at‐homeness extended beyond the hostel itself. During hot summer periods, when air‐conditioning was turned off for energy‐saving purposes, *Bo* regularly walked to a nearby barbershop to “escape the heat”. Although he only had his hair cut there once a month, he visited frequently to cool down and chat with the owner. The barbershop—just a two‐minute walk away and characterised by a small, cosy interior—appeared to offer *Bo* a sense of ease and familiarity that complemented his experience of home within the hostel.

**FIGURE 8:**
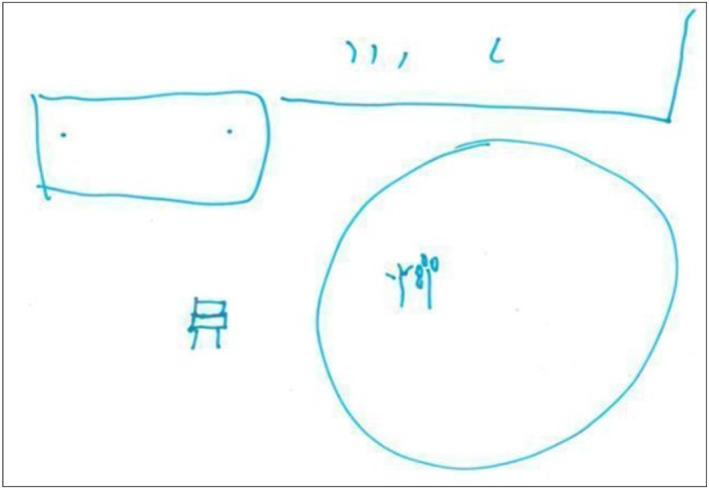
*Timothy's* home as drinking beer near the beach.

**FIGURE 9:**
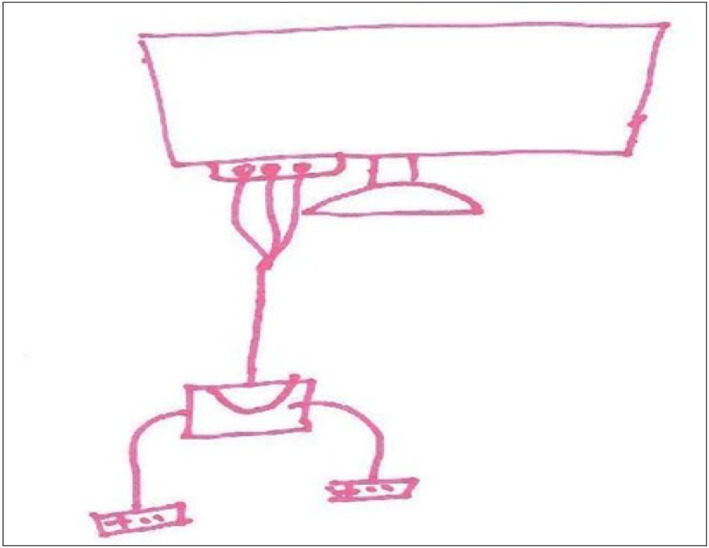
*Donald's* home in PlayStation.

Thirdly, the imaginary of home was also enacted within specific moments in time, when particular activities took place. This dimension of home offers (temporary) spaces in which boundaries and bonds with “people” or place can be configured in ways participants found more ideal. For example, *Timothy*, a 31‐year‐old resident, denied having any mental illness and described being compelled to live in the hostel under a Community Treatment Order. At his parents' home, where he previously lived, his mother often scolded him for work issues. For *Timothy*, therefore, home was not tied to a fixed place but existed in moments when he “*went to the beach at night to drink beer*” (Figure 8), where he could experience “freedom and quietness”. Similarly, some participants located home in moments of recreation (e.g., playing games; Figure 9). *Donald*, a 42‐year‐old resident who had lived in multiple hostels and was about to live by himself soon, described recreational facilities as an essential component of home; without them, he would feel everyday life “*too boring*”.

**FIGURE 10:**
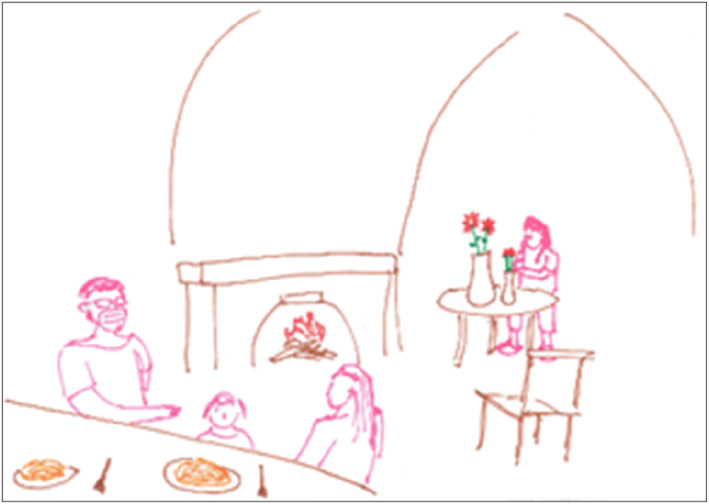
Steven's drawing.

**FIGURE 11:**
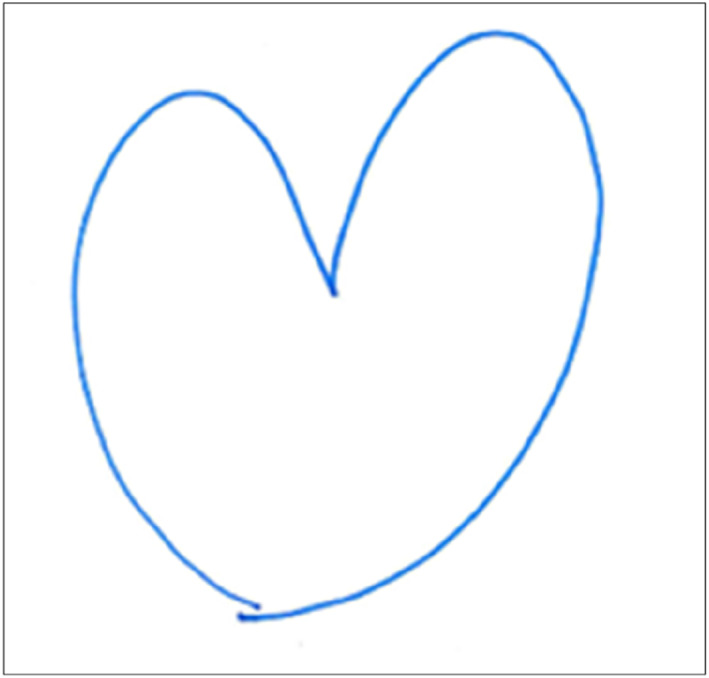
Justin's drawing.

Lastly, it is important to emphasise that the contextualisation of at‐homeness is, in itself, a deeply personal construction. For some participants, all three arenas appeared simultaneously in their imaginaries of home (Figure 10). For others, certain arenas were deliberately excluded or downplayed. For example, *James*, a single man who expressed strong resentment for living in a hostel, portrayed his home as an apartment that permitted only himself. In contrast, *Andrew*, a 54‐year‐old first‐time resident who missed living with his wife and daughter, emphasised that family was the most important aspect of home—“*nothing else, not property or wealth, matters.*” For *Justin*, a 60‐year‐old resident with extensive experience living in residential settings, home could even be reduced to one's body; as he explained when interpreting his drawing (Figure 11), his ideal home was his own health. These variations highlight the multiscalar and deeply subjective nature of home, underscoring the importance of examining the processes through which it is produced.

### 3.2 Conceptualising At‐Homeness

How “people”, materials, and/or activities are positioned within one's imaginaries of home often connects to the underlying pursuits that reflect what the experience of being metaphorically at home means to each individual. Participants' interpretations of their drawings and card‐sorting exercises further refined and deepened the initial seventeen essences of at‐homeness identified through the review of previous literature on home and consumer consultations. Table 1 summarises these core essences of at‐homeness across personal, relational, material and temporal dimensions, illustrating them based on analyses of participant accounts and ethnographic observations.

**TABLE 1:** Interpretations of core essences of at‐homeness based on participant accounts and ethnographic observations.

Quality (%)	Personal	Relational	Material	Temporal
Safety (73%)	Ontological security derived from a meaningful, stable life (e.g., having a fixed income and residence).	Protection from abuse, theft, violation, bullying or discrimination by others (e.g. family, staff, roommates).	Shelter from external dangers and risks (e.g., security measures, CCTV, window grilles).	Freedom from fear of the unknown future (e.g., not knowing the path after leaving the hostel).
Personal space (53%)	Private space for safeguarding personal information (e.g., diagnosis, marital status) from disclosure.	Distance from others and freedom from intrusion (e.g., avoiding nagging or unwanted interaction).	Exclusive use of a physical area within the residence (e.g., a private room, bed or storage).	Undisturbed personal time (e.g., walking in parks, listening to music).
Freedom/Choice/Control (63%)	The degree to which one can choose and act according to personal will.	The ability to define and regulate interactions and relational rules with others (e.g., family, staff) or entities (e.g., the hostel).	Autonomy to select and use facilities, spaces and objects freely.	Freedom to arrange one's own time and schedule (e.g., going out at any time).
Identity (23%)	The roles and titles (e.g., son, resident) that define a person and provide them with a corresponding position, accompanied by rights and responsibilities, within their interactions with people, material objects and time in the home.			
Ownership (25%)	A sense of legitimacy and entitlement (e.g., legal ownership of property) which enables individuals to exercise agency in shaping their relationships with others, the environment and temporal rhythms.			
Warmth (63%)		A relational state of being seen, cared for and emotionally accompanied, where one's needs and existence are recognised and affirmed (e.g., having one's birthday remembered).	A sense of attachment between the individual and their surroundings, where the environment, furniture and facilities align with one's personal needs and preferences.	
Care (53%)		Mutual acts of attentiveness and support among individuals within the home (e.g., greeting, conversing, accompanying, sharing, helping or giving gifts). A common strategy for enacting the relational experience of home.		
Respect (48%)		A sense of equality in interactions with others (e.g., polite and reciprocal treatment). Recognition of one's opinions, preferences and ideas as valued rather than dismissed or ridiculed. A common strategy for enacting the relational experience of home.		
Family relatedness (68%)		Kinship ties grounded in familial or blood relationships. Relationships that resemble familial bonds, formed through intimacy and sustained interaction. An anchor for the relational experience of home, providing continuity and emotional security.		
Environment (42%)			A sense of comfort, aesthetic pleasure and convenience facilitated by the internal and external surroundings of the home (e.g., convenient transportation, sea or mountain views).	
Tidiness/Cleanliness (68%)			A state of order and organisation that enhances one's sense of comfort, stability and control. Environmental hygiene that ensures a healthy and dignified living environment.	
Food (53%)			A basic necessity for survival. A regular routine that structures daily life. A pursuit of quality living, reflected in attention to taste, presentation and sensory satisfaction. A process of selection and creation enacted through both preparing and taking meals.	
Familiarity (27%)		A feeling of belonging and intimacy developed through stable routines, habits and relationships (e.g., living together for years).	Smooth, embodied engagement with one's environment (e.g., knowing which day's meals are best).	A feeling of predictability and continuity naturally developed over time.

*Note:* • % refers to the percentage of interviewees who selected this quality.

Although space does not permit elaboration on each quality in detail, two points are particularly crucial for understanding and transferring this concept into care practice. First, much like the notion of home itself, these essences of at‐homeness may appear deceptively familiar, inviting the risk of projecting one's own assumptions onto others' experiences. Yet, as participant accounts demonstrate, many of these essences are inherently multi‐dimensional. For instance, safety was variously interpreted as a physical condition (e.g., “*the building is secure*” [*Joshua*]; “*my property is protected*” [*Stephen*]), a relational experience (e.g., “*being protected by family*” [*Eric]*; “*having well‐behaved neighbours*” [*Ryan]*), a psychological state (e.g., “*having stable work and income*” [*Larry]*)); or a temporal sense of security, described by some as “*freedom from fear about the future or the unknown*” (*Andrew*). Although some participants emphasised one specific aspect, others explicitly highlighted the multiplicity of the concept. This subjectivity and fluidity in interpreting the essences of at‐homeness caution against assuming a universal meaning and instead highlight the possibilities for these understandings to shift across dimensions.

Another significant finding emerging from the card‐sorting exercises and participants' interpretations is that, although certain qualities (e.g., safety, selected by 73% of participants and family relatedness, 68%) were more frequently selected to represent participants' feelings of home, none proved universally indispensable. The way residents organised these essences to construct their sense of at‐homeness varied markedly across individuals. This diversity cautions against imposing a common or hierarchical model of human needs to define or evaluate at‐homeness in residential settings. Rather, the findings underscore that at‐homeness is inherently individualised and situational, shaped through subjective prioritisation and contextual negotiation. This, again, directs attention to the constructive processes through which at‐homeness is enacted, as explored in the following section.

### 3.3 Constructing At‐Homeness

The preceding findings demonstrated that subjectivity permeates the contextualisation and conceptualisation of at‐homeness. Yet, how do these subjective understandings take shape and become negotiated with reality, particularly among residents of care settings who have often experienced illness, suffering or major disruptions in life? For the vast majority of participants, the contexts and essences of at‐homeness were not regarded as parallel or equally weighted. Although certain constructions appeared to emerge without explicit reflection, most participants displayed an identifiable internal logic guiding how they selected, prioritised or downplayed particular elements in constructing their sense of at‐homeness, reflecting core practices in this process: self‐appraisal, temporal integration, relational attunement and socio‐cultural grounding.

#### 3.3.1 Self‐Appraisal of Personal Needs

Within these narrative logics, the first integral consideration concerns an awareness/appraisal of one's core needs, that is, what is essential versus supplementary for feeling at home. Such prioritisation was often grounded in participants' reflections on their life stage, alongside factors such as diagnosis and residential experience. For example, *Justin*, a 60‐year‐old man with schizophrenia who has lived in various hostels for over 2 decades, described his home as his healthy body, explaining that “*at my age, health is the most important, so the hostel is good enough—I can have friends there and don't need much money.*” Considerations of ageing‐related needs were echoed in the accounts of many older residents and subsequently informed their residential decisions. At the second ethnographic site, *Mary*, in her 50s, described feeling at home living in the hostel and repeatedly declined staff assistance to apply for public housing, citing health conditions, limited savings, imminent retirement and the absence of family support.

This assessment might also be shaped by long‐term familiarity with hostel living, which several participants described as “*something I'm used to*”. Although *Justin* identified freedom as an important quality of at‐homeness, he defined freedom as the ability to “*come and go freely*” and agreed with most institutional rules, such as supervised self‐care and medication management. These regulations, in his account, helped to “*prevent infectious diseases*”, thereby contributing to a sense of safety—another essential quality underpinning his conceptualisation of at‐homeness.

In contrast, younger participants rarely foregrounded health or care needs when constructing at‐homeness, instead reflecting ongoing negotiations between independence and relational attachments. *Nancy*, who had lived in the hostel for over two years since turning 18, entered residential care voluntarily. She explained that her family had already expended considerable resources supporting her schizophrenia and that, having “*grown up*”, she felt a responsibility to live independently to reduce their burden. Her ideal home was imagined as a “*self‐sufficient cabin in the forest*”, where she could live alone whilst enjoying safety and personal space.

For another participant, *John* (28 years old with depression), the pursuit of independence was more ambivalent. On the one hand, he described family co‐residence as emotionally fraught—“*living with family makes me relapse more easily.*” On the other hand, after 6 or 7 years of living independently, he experienced solitude as “*too cold*”. As a result, although *John* continued to locate his idea of home within the context of family, he emphasised that such a home must be premised on a sufficient level of care, respect and warmth.

#### 3.3.2 Temporal Integration

For some participants, this appraisal of personal needs may stem from an intention to integrate their most fulfilling/cherished experiences and/or the deep losses/regrets of the past or present into their imaginary home, which to some degree carries a sense of ideality or hope for the future. For example, *Linda*, a 27‐year‐old resident who had previously lived independently, had been living in the hostel for over 6 months at the time of the interview. Following her admission, her only close family—her sister's family—provided frequent support, such as buying clothes and bringing food. *Linda* imagined at‐homeness as living together with her sister, prioritising family relatedness and sharing of food and safety. She explained that this construction was grounded in her current lived reality, as she regularly visited her sister and found joy in spending time with her two nieces, which also represented her most desired direction for life after leaving the hostel.

For others, past experiences of suffering played a decisive role in shaping what they regarded as essential to at‐homeness. *Katherine*, a 54‐year‐old woman, described having endured prolonged inequality and humiliation within her previous marriage, which eroded her sense of safety and identity. As she reflected, “*After marriage, that feeling [of safety] disappeared—there was no place where I could stand on my own*.” Following her divorce, the onset of depression and anxiety and psychiatric hospitalisation, *Katherine* had been living in the hostel for over 2 years and was awaiting public housing allocation. For her, at‐homeness was constructed as a “*stable*” place, one fundamentally defined by respect and safety.

Furthermore, some participants' constructions of at‐homeness reflected a more integrative fusion of past, present and future. For example, *Stephen,* a man in his 50s, did not favour hostel living and instead depicted his ideal home as living independently in a suburban area. As he explained, this idea was grounded in a long history of self‐reliance: *“I've always lived on my own. No problem at all, really.*” When drawing and reflecting on home, however, *Stephen* also recalled childhood memories of being cared for by his parents: “*My mum and dad used to be there. They looked after me; I didn't have to think about anything.*” He paused before adding quietly, “*But they have already passed away.*” Now, he only visited his siblings occasionally. *Stephen*'s construction of at‐homeness seems to have drawn simultaneously on past experiences of independence, the present absence of close family support and a future‐oriented plan for post‐discharge independent living that he had begun to discuss with hostel staff.

#### 3.3.3 Relational Attunement

Although personal needs were central, many participants who emphasised the relational dimension of at‐homeness also considered the needs of significant others within the home and/or how a proper bond could be maintained. Such considerations often interacted with the temporal constructions of at‐homeness, whereby participants highlighted activities (e.g., shared meals or helping with household tasks) to demonstrate care and respect and to balance personal and relational needs.

A less frequently acknowledged, but equally important, point is that these relational considerations also influenced how the material dimension of at‐homeness was organised, that is, what was included, excluded and how objects or spaces were positioned to serve relational purposes. For example, *Steven,* a 31‐year‐old resident with schizophrenia, described co‐residing with his mother as offering “*the least personal space*”, but had nonetheless chosen hostels located closest to her home on two separate applications. In his drawing (Figure 10), *Steven* integrated both relational imaginaries of his mother and a future family into his ideal of home. He placed himself on the left, his imagined wife and child in the centre and his mother in the background, sitting and engaging in her favourite pastime, flower arranging. This distance between himself and his mother, as he explained, represented an ideal balance—one that allowed mutual care without excessive intrusion. This helps explain *Steven*'s expressed satisfaction with the hostel, which provides an appropriate degree of separation from his family. *John* articulated this more explicitly, describing the hostel as a “*buffer*” from his mother's constant nagging.

Further, constructing the material dimension of at‐homeness around family needs was also evident in *Ryan's* account. *Ryan*, a 50‐year‐old resident, had jointly applied for public housing with his mother and had been allocated an apartment several months prior to the interview. As he anticipated moving out of the hostel, his depiction of an ideal home was largely organised around how the future living environment could accommodate his mother's needs. For instance, he emphasised tidiness/cleanliness as a core essence of at‐homeness, explaining that “*If the home looks like a rubbish house, my mum won't feel comfortable living there.”* He also stressed the importance of ensuring that his mother could “sleep well”, stating that he would avoid using bunk beds and instead sleep on a sofa bed himself. In this way, *Ryan*'s construction of at‐homeness was shaped less by his own material preferences than by a deliberate prioritisation of his mother's wellbeing.

#### 3.3.4 Socio‐Cultural Grounding

In terms of the socio‐cultural influences, an awareness of the structural and environmental realities was also evident in many participants' constructions of at‐homeness. For instance, the selection and arrangement of material objects within the home imaginaries often reflected not only personal or family needs but also participants' understandings of the local living environment. A previously mentioned example is *Philip's* emphasis on including a clothes dryer in his imagined home to address humidity (Figure 5). A more representative example of this tendency was demonstrated by how participants envisioned the ideal size of their homes. Many participants who mentioned this aspect imagined living spaces of no more than 500 square feet; for example, *Lisa* remarked, “*I think 300 square feet would be comfortable*”, whereas *Elizabeth* added, “*Even 200‐something square feet is enough for five people.*” Such perceptions are closely related to Hong Kong's notoriously high housing prices and cramped living conditions—realities that most participants had experienced both in hostels and the wider community. This suggests that the construction of at‐homeness, even within idealised imaginaries, may remain grounded in what participants perceive as socially and materially realistic.

Further, an appreciation of cultural traditions and norms also permeated participants' constructions of at‐homeness. For example, *David* emphasised that the design of a home should conform to the principles of Chinese feng shui (a traditional geomantic system concerned with harmonising spatial arrangement and energy flow to promote wellbeing and balance). As he explained, “*My room does not have to be big, but should be well arranged. For instance, the toilet cannot face the main door—because of feng shui.*” In the relational dimension of at‐homeness, a particularly salient example was the expression of filial piety, a deeply valued moral ideal in Chinese societies. The image of parents appeared not only in nostalgic recollections of childhood homes but also in participants' imagined or future versions of home, even among residents in later life when their parents might lack adequate care (e.g., in *Ryan*'s accounts).

*Katherine* (54 years old) articulated this cultural emphasis more explicitly. She imagined home in a more future‐oriented sense, defining it as a stable place in contrast to her past unhappy marriage and one that would allow her to care for her ageing mother. As she stressed, “*Her (mother's) health is getting worse. I just hope I can still take care of her before she passes, so I won't have any regrets*.” Locally grounded moral beliefs are often consciously or unconsciously woven into personal constructions of at‐homeness.

## 4 Discussion

**FIGURE 12:**
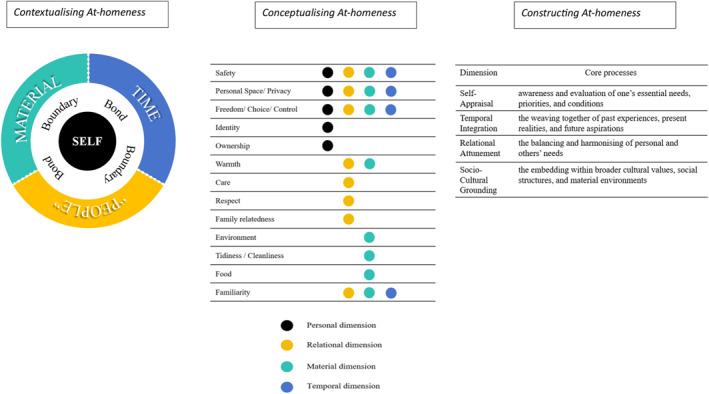
An Integrative framework of At‐homeness for Residential Care.

The intention to cultivate some level of being metaphorically at home has become deeply embedded in the modern organisation of residential care, as reflected in the widespread practice of naming such facilities as “home”. This aspiration has propelled the growing conceptual prominence of at‐homeness as a lens for extending the rich theorisation of home into the domains of health and social care. Drawing on literature review and qualitative analyses, this paper examined the contexts, meanings and constructive processes of at‐homeness from residents' own perspectives. Together, these analyses form the basis of an integrative framework for understanding narratives of at‐homeness in residential care (Figure 12). To understand the distinctiveness and limitations of this framework, several points warrant emphasis:

1. **Integration**: The framework integrates the contextualisation, conceptualisation and construction of at‐homeness. These elements should not be viewed in isolation; rather, they constitute an integrated system of investigation on at‐homeness. By doing so, it brings contemporary home scholarship into closer dialogue with care literature, consolidates prior conceptual developments of at‐homeness and foregrounds residents' personal construction as an interpretive lens through which the negotiation of key domains and qualities of at‐homeness—such as inclusion/exclusion or prioritisation—can be examined.
2. **Multiplicity and Fluidity:** Although the framework identifies multiple domains, elements and constructing processes, our analyses demonstrate that these components are neither universally mandatory nor defined by a singular, fixed meaning. The framework should therefore be understood in light of the multiplicity and fluidity of home (Duyvendak 2011), underscoring the importance of attending to individual narratives instead of attempting to standardise or predefine at‐homeness. This will be discussed in detail in the implications section.
3. **Contextual and Multiscalar Flexibility:** Although the concept of at‐homeness arises primarily in research on residential care, this framework does not confine it to experiences within such settings (e.g., by asking participants to photograph or describe how the hostel “feels like home” [Cater et al. 2022; Chinn et al. 2024; Wada et al. 2020]). Rather, it integrates the flexibility for residents to reflect on how their current residence is positioned within their broader constructions of at‐homeness. In doing so, the framework accommodates the multiscalar nature of home (Blunt and Dowling 2022) and recognises that “non‐home as a choice” (Boccagni and Miranda Nieto 2022) in the construction of at‐homeness.
4. **Emancipation:** This framework adopts an emancipatory approach to researching at‐homeness. It prioritises residents' direct accounts in each component, rather than relying primarily on researchers' inductive interpretations of lived experience as the main pathway to understanding home and at‐homeness. The findings demonstrate residents' capacity to contemplate and articulate the concept in depth, which has long been recognised as inherently challenging to define (Buckley 1971). Importantly, the framework provides a relatively structured method—supported by interview techniques such as drawing and card‐sorting activities—to elicit residents' own constructions of at‐homeness with greater clarity and authenticity.
5. **Sensitivity:** Finally, this framework of at‐homeness should be considered with sensitivity to socio‐cultural variation and cognitive diversity. The core meanings of at‐homeness may be constructed differently across cultural contexts and no single framework can exhaust these nuances. Although the thirteen at‐homeness elements synthesised in this framework may serve as analytical reference points, residents should be invited to articulate additional qualities that extend beyond the framework. Furthermore, for residents with cognitive impairments, engaging with at‐homeness at this level of abstraction may present challenges. Nevertheless, efforts to elicit personal accounts should remain prioritised—potentially with appropriate methodological adaptations—rather than relying exclusively on proxy responses or researcher interpretation.

Examining at‐homeness within the context of transitional mental health residential care also yields important insights into the influences on the construction of at‐homeness. First, compared with research on older adults in long‐term care, life‐course transitions emerged as a particularly salient influence. In elderly residential care, constructions of at‐homeness are often closely tied to bodily decline, functional capacity and security needs—for instance, ensuring safety, maintaining autonomy or managing daily activities despite physical deterioration (Cooney 2012; Lindahl et al. 2003; Saarnio et al. 2016). Such concerns were echoed among some older participants in this study, particularly those with limited family ties. In contrast, younger and middle‐aged participants rarely foregrounded their own care needs; instead, their constructions of at‐homeness were more strongly oriented towards independence, relational attachment (e.g., marriage and family), and, in some cases, identities as future caregivers rather than care recipients.

In addition to life‐course positioning, constructions of at‐homeness were also shaped by residents' interpretations of illness and its social consequences. In studies of older adults, illness and disease are more often framed as normative life‐stage conditions and at‐homeness tends to be conceptualised as an optimal residential experience achievable despite bodily decline (Öhlén et al. 2014). In contrast, mental illness in this study was embedded in more contested identities and past relational ruptures, producing more complex effects on at‐homeness constructions. For some participants who denied having a mental illness or experienced involuntary admission, illness was associated with loss of autonomy, leading them to prioritise solitary living as the only authentic form of home. For others, traumatic experiences linked to illness onset (e.g., domestic violence) shaped temporal constructions of at‐homeness, with preferred qualities of at‐homeness articulated in contrast to past suffering.

Analyses also offer a valuable opportunity to discuss the relationship between at‐homeness and temporality. These two concepts may appear opposed, as some humanistic geographers and sociologists have often regarded place attachment as “good” and equated mobility with “uprootedness and social disintegration” (Gustafson 2014, 38). For example, Harris et al. (2020) highlighted the reproduction of stigma and precarity through material interactions in temporary accommodation, whilst Padgett (2007) concluded that transitional housing for people with mental health conditions offers limited support for ontological security and the benefits of “home”. However, in this study, although many participants articulated ideal homes as imagined or future‐oriented destinations, this does not imply that transitional accommodation has only negative implications. Some participants actively chose to return to the same hostel, whereas others constructed at‐homeness as distributed across multiple sites (e.g., hostels and parental homes). Even for those who located at‐homeness primarily in a post‐discharge future, transitional accommodation may function as a meaningful temporal node—a place of waiting and preparation (e.g., whilst awaiting public housing), facilitated by its relative affordability compared to rental markets. These findings point to a spatially and temporally broadened understanding of at‐homeness, in which temporary settings are not merely deficient spaces but integral moments in the ongoing construction of at‐homeness.

Furthermore, by highlighting the role of socio‐cultural grounding in constructing at‐homeness, this paper extends research on at‐homeness, which has predominantly emerged from Westernised conceptualisations of home (Hoolachan 2020). Culture‐specific nuances in constructing at‐homeness are evident in this study, exemplified by the prominent emphasis on filial piety—an element that has rarely featured in at‐homeness research within or beyond elderly care in Western contexts. Nonetheless, it is important not to over‐culturalise these findings. Although filial piety and strong family bonds are often regarded as more deeply embedded in Chinese societies than in Anglo‐European contexts (Cui et al. 2025), cultural values alone may not deterministically shape constructions of at‐homeness. Micro‐level relational dynamics (e.g., intra‐family relationships) and macro‐level structural factors (e.g., policy contexts), may exert significant influence. For example, to encourage younger families to care for ageing parents, Hong Kong has introduced priority public housing schemes for multigenerational households (Hong Kong Housing Authority 2019), substantially shortening waiting times. Such policies may further legitimise and reinforce family‐centred imaginaries of home and care.

### 4.1 Implications for Practice

The findings offer important implications for practice in residential care. First, this structured exploration of residents' narratives of at‐homeness holds particular importance for the field of residential care. Such exploration itself can have an empowering effect on multiple levels. For the residents, gaining clarity about how they construct their own at‐homeness also means developing a deeper understanding of what constitutes their optimal spatial experience and how this aligns with their broader aspirations for living. It is also a reflective process through which individuals recognise that within their imagined home, the inclusion or exclusion of particular people, materials or activities represents their own choices, including the choice not to choose. This awareness‐raising process can play a powerful role in facilitating residents' transformation from passive recipients of care to active agents in shaping their lived environments. Indeed, many interviewees expressed appreciation at the end of the interviews, noting that the discussion had helped them clarify their orientations towards dwelling. More broadly, such an approach challenges the prevailing tendency in social care to position service users as passive beneficiaries (Cui et al. 2019), redirecting attention in at‐homeness‐related interventions beyond environmental or professional enhancement towards recognising and supporting the person's own active role in meaning‐making and homemaking.

Further, exploring how and why particular contexts or essences of at‐homeness are articulated and positioned carries significant therapeutic potential. Such inquiry can illuminate the values, ideologies and emotional orientations that underpin an individual's meaning‐making process, offering deeper insight into how residents experience and interpret their living environments. This understanding may assist clinicians during the service‐matching process in identifying potential mismatches between a resident's personal values and the institutional ethos. It can also help uncover underlying tensions or sources of unease in residents' everyday lives—for instance, when narratives of at‐homeness are dominated by nostalgic memories or attachments to past certainties, which may signal feelings of threat or disorientation in the present (Reinders and Van Der Land 2008). Additionally, some residents' constructions may reveal irrational or unconscious cognitive patterns that could benefit from therapeutic dialogue aimed at gradual reframing and adaptation. Thus, embedding the narrative analysis of at‐homeness—a concept that bridges psychological, relational, environmental, temporal and sociocultural dimensions—into residential care practice opens up new clinical and developmental possibilities. The integrative framework of at‐homeness proposed in this study provides a conceptual foundation for developing such interventions, inviting further co‐design initiatives that incorporate residents' and stakeholders' voices into care innovation.

Last but not least, analyses of this constructive process foreground the dynamic and evolving nature of at‐homeness. It suggests that constructions of at‐homeness may shift in response to changes in personal circumstances (e.g., life course transitions), relational configurations and temporal conditions. Aligning with the critical perspective on home, this indicates that constructing at‐homeness is an ongoing process shaped not only through cognitive and emotional meaning‐making but also through the everyday practices by which individuals attempt to bridge idealised imaginaries of home with situated social realities. Importantly, our framework provides both conceptual and methodological clarity for future research into the homemaking and unmaking practices of residential care. This line of inquiry is critical for the sector. Although residential care systems often prioritise measurable care outcomes (e.g., recovery outcomes in mental health residential care), understanding how people *reside* well should be recognised as a central—and uniquely fitting—focus of such services, one that requires collective efforts from organisations, practitioners, and, crucially, residents themselves.

## Author Contributions

**Jialiang Cui:** conceptualization, methodology, investigation, validation, formal analysis, supervision, data curation, writing – original draft, writing – review and editing, funding acquisition. **Jialing Wu:** software, investigation, validation, formal analysis, project administration, writing – review and editing.

## Funding

The work described in this paper was fully supported by a grant from the Research Grants Council of the Hong Kong Special Administrative Region, China (Project No. CUHK 24619023).

## Ethics Statement

Ethical approval was obtained from the ethical committee at the Chinese University of Hong Kong (Reference No. SBRE‐22‐0195).

## Conflicts of Interest

The authors declare no conflicts of interest.

## Data Availability

Research data are not shared.
